# Vanished Without a Trace: A Middle‐Aged Man With Vanishing Bile Duct Syndrome—A Case Report

**DOI:** 10.1155/crgm/4371217

**Published:** 2026-06-30

**Authors:** Edwin Mendoza, Alan Jurado, Virali Gulla, Gian Rodriguez Franco, Osvaldo Padilla, Sherif Elhanafi, Luis Chozet, Tamis Bright

**Affiliations:** ^1^ Department of Internal Medicine, Texas Tech University Health Sciences Center, El Paso, Texas, USA, ttuhsc.edu

**Keywords:** drug-induced liver injury, hyperlipidemia, vanishing bile duct syndrome

## Abstract

Vanishing bile duct syndrome, VBDS, is a rare disease that is poorly understood and is a term used to loosely describe a group of disorders associated with the progressive destruction of intrahepatic ducts. This syndrome ultimately leads to cholestasis. There are many potential etiologies for this disease including primary biliary cholangitis (PBC), primary sclerosing cholangitis (PSC), autoimmune diseases, medications, genetic abnormalities, infectious causes, or neoplastic disorders. The diagnosis consists of both clinical and histological findings found via liver biopsy. We present a rare case of VBDS in a 41‐year‐old male likely attributed to multiple herbal supplements in the setting of severe hyperlipidemia. This case highlights the importance of recognizing supplement‐related liver injury and unusual metabolic associations.

## 1. Introduction

Vanishing bile duct syndrome (VBDS) is a rare cholestatic disorder characterized by progressive loss of intrahepatic bile ducts found via liver biopsy. VBDS is rare and epidemiologic data are presented mostly indirect; for example, VBDS proportions are among bile duct diseases or VBDS case series by cause, rather than classic population‐based prevalence rates. Nevertheless, there are some examples of the prevalence of this disease such as those described in Bonkovsky et al. In a prospective study utilizing data from the drug‐induced liver injury network (DILN), of 363 patients that had undergone liver biopsy, only 7% of patients had histological evidence of bile duct loss [1]. Additionally, in a case report by Salman et al., they describe a rare case of VBDS in a patient with Hodgkin’s lymphoma (HL) and state that VBDS has been reported in approximately 40 cases of HL [2]. VBDS can present with a variety of symptoms such as jaundice, pruritus, fatigue, and weight loss [3]. Patients can also experience clinical manifestations of cholestasis such as hyperlipidemia, xanthelasmas, and gallstone formation [3]. The laboratory workup typically shows direct hyperbilirubinemia, elevated alkaline phosphatase, elevated gamma‐glutamyl transferase, and may even show elevated liver enzymes [3]. This unique case highlights a rarely described case of VBDS in a patient who took multiple over‐the‐counter supplements in the setting of severe hyperlipidemia. This disease is associated with certain medications, autoimmune conditions, genetic conditions, graft‐versus‐host disease, and viral pathogens. There is a rising incidence of herbal and dietary supplements causing drug‐induced liver injury (DILI) [4]. There is a lack of literature on VBDS linked to over‐the‐counter supplements and severe hyperlipidemia. Our objective in this case report is to present an atypical presentation of VBDS potentially associated with multisupplement exposure and profound hypercholesteremia.

## 2. Case Report

A 41‐year‐old male patient with a past medical history of Type II diabetes mellitus not on any medication, a recent history of transaminitis with hyperbilirubinemia status post cholecystectomy and endoscopic retrograde cholangiopancreatography (ERCP), hypercholesterolemia came to the ED after being sent by his endocrinologist for LDL‐apheresis. The patient’s outpatient endocrinologist had planned to prescribe evolocumab 140 mg subcutaneously every 2 weeks to lower the cholesterol levels but decided to have the patient come into the hospital instead. The patient stated that his symptoms began 3 months before admission and endorsed abdominal pain with jaundice at the time. The patient stated he had a cholecystectomy at that time and nonobstructing stones were removed. It was during that time the lipid profile was found to be significantly elevated, and the patient was referred to endocrinology.

On admission, the patient endorsed diffuse itching with pale stools, and physical examination was remarkable for jaundiced conjunctiva without any asterixis. On laboratory workup, he was found to have elevated cholesterol greater than 1200 mg/dL (normal 120–200), and LDL levels at 1138 mg/dL (normal 50–100). Additionally, the patient had an elevated ALT of 252 unit/L (normal 0–50), AST 183 unit/L (normal 17–59), and an alkaline phosphatase of 1703 unit/L (normal 38–126) (Table 1). A central line was placed in the emergency department, and nephrology was immediately consulted for plasmapheresis due to hyperlipidemia being suspected for causing the liver injury. Gastroenterology was also consulted for the transaminitis, and they recommended an interventional radiology consult for a liver biopsy to find the etiology of the injury. Relevant hepatobiliary disease workup such as antimitochondrial antibodies (AMA), antismooth muscle antibodies (ASMA), IgG antibodies, and hepatitis panel were negative. Genetic testing for hyperlipidemia such as lipoprotein A or ApoB was not performed, and the patient denied any familial hypercholesteremia. Additionally, the patient did not have any features to suggest familial hypercholesteremia but did have jaundiced conjunctiva on physical exam. Additionally, the patient did not have any evidence of immunosuppression such as HIV or any malignancy which is important causes associated with VBDS. The R factor was 0.4 on admission, indicating a cholestatic injury pattern. The patient had an ultrasound of the abdominal artery done as an outpatient 4 days before admission which showed a common bile duct measuring 4 mm, and no intrahepatic or extrahepatic bile duct dilation was noted.

**TABLE 1:** Laboratory investigations at admission.

Lab parameter (units)	Results at admission	Reference ranges (units)
Hemoglobin (g/dL)	11.7	12–15
Hematocrit	36.3	36–47
Total leukocyte count (cells/uL)	12.56	4.5–11.0
Platelet count (units)	468	150–450
MCV	100.3	82–98
Sodium (meq/L)	135	135–145
Potassium (meq/L)	4.0	3.5–5.1
Chloride (meq/L)	102	98–107
Calcium (meq/L)	9.4	8.4–10.2
Blood urea nitrogen (mg/dL)	13	7–17
Serum creatinine (mg/dL)	0.8 (eGFR 113)	0.52–1.04
Serum glucose (mg/dL)	90	74–106
Carbon dioxide	15	22–30
Serum total bilirubin	17.5	0.2–1.3
Alanine transaminase (U/L)	252	0–35
Aspartate transaminase (U/L)	183	14–36
Alkaline phosphatase (U/L)	1703	38–126

**FIGURE 1:**
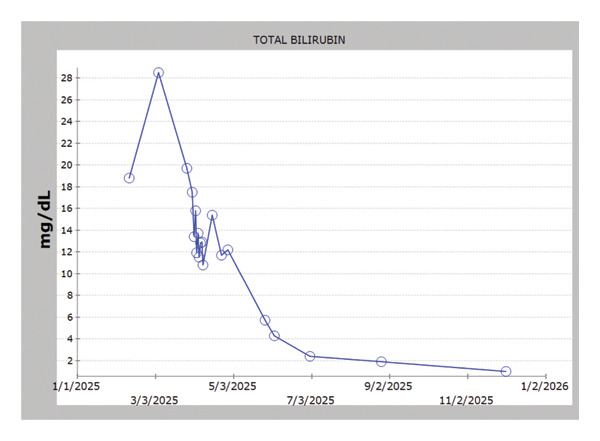
Bilirubin levels from initial presentation to months later.

**FIGURE 2:**
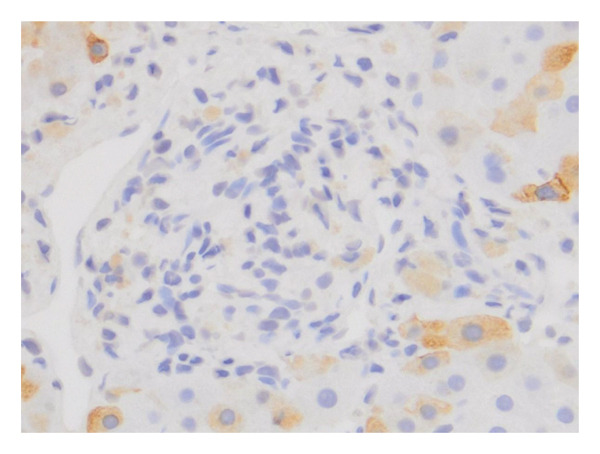
CK IHC stains fail to show any biliary ducts in the portal triad areas (the staining see is nonspecific staining in hepatocytes).

**FIGURE 3:**
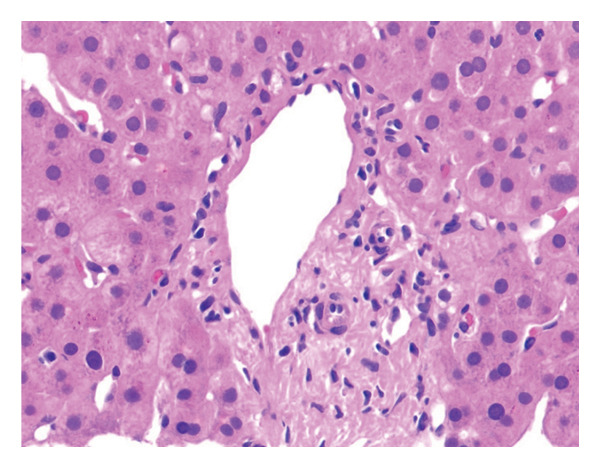
The H&E shows a portal triad area without bile ducts (ductopenia), which affected greater than 50% of portal triads within the entire biopsy.

Repeat bilirubin levels demonstrate an improvement seen in Figure 1. Liver enzymes were not able to be plotted, but AST was initially 183 Unit/L with levels improving to 63 Unit/L 8 months later. Additionally, ALT levels were 252 Unit/L on admission and later improved to 130 Unit/L 8 months later. The liver biopsy showed an intact hepatic architecture with periportal intrahepatic cholestasis and ductopenia (greater than 50% of portal triad areas showed biliary duct loss) (see Figures 2 and 3) Some of the portal triad areas also showed some mild degree of chronic portal hepatitis and spotty necrosis (grade 1; Batts Ludwig classification). No cholangitis was clearly identified. Other laboratory markers such as ceruloplasmin 66 mg/dL (normal 14–30 mg/dL) and alpha‐1‐antitrypsin 200 mg/dL (normal 83–199 mg/dL) were mildly elevated. Multiple imaging studies such as MRA of the abdomen with and without contrast and ultrasound with Doppler were inconclusive and did not illustrate any pathology related to her laboratory abnormalities.

Interestingly, the patient had previously been on 2 diabetes medications in the past, which were sitagliptin 100 mg daily and glipizide 2.5 mg daily; however, the patient was motivated to implement dietary measures so that he could discontinue his medications. In these efforts, he had a strict ketogenic diet and had started taking multiple herbal supplements, as many as 15 types a day. The patient provided a picture of these medications which included vitamin B1 250 mg, Maca 500 mg, *Moringa Oleifera* 1000 mg, acetyl L‐carnitine HCL 400 mg with alpha lipoic acid 200 mg, nitric oxide 10 mg, DHEA 50 mg, Zinc 50 mg, dandelion root, Men’s multivitamin, ashwagandha, elderberry 50 mg, fish oil minis 1400 mg, methylated folate, lycopene vitamin D, vitamin D complex, tribulus muira puama, Asian ginseng, niacin, and Icelandic Kelp. The patient had a total of four plasmapheresis sessions during the inpatient stay, but after two days of no sessions, his LDL began increasing again, from 124 to 165. However, after his fourth session, his cholesterol and LDL levels began to decline, and the patient was ultimately discharged with gastroenterology and endocrinology follow‐up.

After one week from being discharged from the hospital, the patient’s cholesterol and LDL levels rose again to 437 and 381, respectively. Endocrinology began inclisiran 284 mg every 3 months to lower the patient’s LDL levels which, after a 1 month follow‐up, did significantly lower his LDL levels to about a 50% reduction, 169 mg/dL LDL.

## 3. Discussion

VBDS refers to a rare cholestatic disease in which the intrahepatic bile ducts in the portal regions progressively degenerate and disappear, referred to as ductopenia, resulting in cholestasis. Typically, greater than 50% loss of intrahepatic bile ducts is seen on histology [5]. Some potential causes of this rare disease include autoimmune conditions, infections, neoplasms, genetic diseases, and even medications. Our patient had biopsy‐confirmed VBDS, likely secondary to multiple supplements being consumed at the same time. This represents a rare case of VBDS being caused by medications in combination that have been reported on their own to cause this disease. Given the broad differential, being able to identify the cause of VBDS is essential, as both management and prognosis are determined by inciting etiology.

The pathophysiology of this disease is poorly understood. Some proposed theories include increased exposure to toxic bile salts due to impaired defenses of the epithelium or direct injury to cholangiocytes after bile excretion [6]. Autoimmune conditions are one of the most common causes of VBDS, diseases such as primary biliary cholangitis (PBC) and primary sclerosing cholangitis, through T cell‐mediated destruction of bile ducts, cause cholestasis. Systemic autoimmune diseases, graft‐versus‐host disease following hematopoietic stem cell transplantation, infectious etiologies such as cytomegalovirus (CMV), Epstein–Barr virus, and human immunodeficiency virus; and even paraneoplastic diseases such as HL have been implicated in VBDS [2]. Therefore, it is important to conduct a comprehensive evaluation of patients presenting with VBDS, as there are many nondrug‐related causes to consider. Workup of autoimmune and viral etiologies causing VBDS in our patient was unremarkable with Epstein–Barr, CMV, and autoimmune markers being negative.

Given an extensive workup that did not reveal evidence of autoimmune conditions, infection, or malignancy, supplement‐induced injury was the most likely etiology in our patient. Beyond autoimmune conditions, many medications have also been implicated in VBDS which include antibiotics such as macrolides, penicillin, cephalosporins, antipsychotics, and nonsteroidal anti‐inflammatory drugs [7]. The medications our patient took have been shown in the literature to cause DILI. In a systematic review and analysis of case reports and case series by Wasuwanich et al., 59 cases of confirmed VBDS demonstrated that antibiotics, particularly beta‐lactams, were the most commonly linked drug to VBDS onset [8]. Some of the medications such as ashwagandha, tribulus terrestris, high‐dose or sustained‐release niacin, and multivitamins with high‐dose vitamin A have been shown to cause DILI. Niacin has been shown to cause DILI when consumed in high doses > 1000 mg/day sustained release forms but has not been shown to cause VBDS. We are unsure if our patient consumed high‐dose niacin formulation. DHEA and ashwagandha have also been shown to cause mild transaminitis but have not been implicated in VBDS. Although many of the medications consumed by our patient have been associated with DILI, direct causation of VBDS has not been clearly established. In contrast to previously reported cases where a single offending agent is identified, this case is notable for concurrent exposure to multiple supplements with hepatotoxic potential, raising concern for a cumulative or synergistic effect leading to bile duct injury. When reviewing the list of the medications our patient consumed, it is likely that these medications in combination contributed to VBDS.

Recent case reports depict the heterogeneity of VBDS presentations. For example, in a case report by Bak et al., they described a 29‐year‐old female with no reported past medical history who came in with symptoms of a cold and subsequently developed severe liver injury after taking the medication pelubiprofen [9]. Similar to our patient, she developed pruritic symptoms and viral, along with autoimmune markers that were negative. In addition, liver biopsy results also demonstrated a paucity of intralobular bile ducts which is what our patient experienced. In contrast, our patient did not experience the need for a liver transplant which unfortunately the patient described by Bak et al. did. In another case report by Ayyad et al., they detailed the clinical course of a 58‐year‐old Hispanic female with HIV on bictegravir/emtricitabine/tenofovir alafenamide who presented with a 3‐month history of cyclical fevers, nausea, vomiting, and anorexia occurring every 15 days with a persistently low CD4 count despite an undetectable viral load [10]. Investigations revealed liver enzymes peaking during fever episodes to levels similar to our patient’s in the 150 s·U/L. Interestingly, Epstein–Barr PCR levels were elevated at 90,490 IU/mL. Ultimately, VBDS was confirmed via liver biopsy showing ductopenia and bile duct paucity. Our patient’s presentation was similarly difficult to pinpoint the etiology of VBDS, as multiple factors were present. However, our patient did present with much higher levels of bilirubin compared to the patient presented by Ayyad et al. which was consistent with the lack of pruritus or jaundice. Finally, another case report by Salman et al. described a 53‐year‐old female with no preexisting liver disease who presented with jaundice, pruritus, vomiting, and diarrhea [2]. After diagnosing HL with bone marrow and excisional lymph node biopsy, and ruling out viral causes of elevated liver function tests, a liver biopsy revealed VBDS. A similar case report of HL causing VBDS was also reported by Bakhit et al. [11]. As shown by recent literature depicting these three cases of VBDS, the presentation has many overlaps but also some differences which emphasizes the importance of clinicians considering other causes leading to VBDS besides medications. Compared to these cases, our patient’s presentation was distinguished by the absence of systemic symptoms suggestive of malignancy or infection and by exposure to multiple supplement uses as the most plausible trigger.

There are many medications that have been implicated in causing liver injury from dietary and herbal supplements. Typically, the presentation of this supplement‐induced injury may follow a pattern of marked elevations in serum aminotransferase levels but no or minimal increases in alkaline phosphatase values [12]. This pattern of injury is atypical to what our patient experienced with elevated alkaline phosphatase levels and moderately elevated liver enzyme levels. A diagnostic challenge that comes when suspecting dietary and herbal supplement‐induced liver injury is that many supplements may contain undeclared ingredients or even be contaminated with unintended ingredients, leading to further potential for liver injury [4]. DILI is a diagnosis of exclusion which entails obtaining a detailed history, obtaining appropriate blood work, and performing hepatobiliary imaging. In addition, when enzymes remain elevated despite removal of the suspected agent, obtaining a liver biopsy is essential for diagnostic and prognostic purposes [13]. Our patient went through a similar workup, ultimately reaching the diagnosis of VBDS. As proposed by Lubarska et al., legislation should be introduced to have mandatory testing for herbal and dietary supplements which would help in choosing the appropriate therapy [14].

Interestingly, our patient presented with extremely elevated cholesterol levels and LDL levels. Elevations in both cholesterol and LDL > 1000 mg/dL have not been implicated in causing VBDS. Instead, cholestasis itself leads to impaired bile excretion and has been shown to cause secondary hyperlipidemia [15]. Our patient did not have any family members with a similar lipid profile which is seen in familial hypercholesterolemia. Additionally, our patient did not have any features of xanthomas, premature coronary artery disease, or pancreatitis that can be seen with extremely elevated lipid profiles. It is possible that the etiology behind the hyperlipidemia is multifactorial and includes extreme ketogenic diets and supplemental drug exposure causing cholestasis, both of which affected the patient.

Current management of VBDS is directed at addressing the underlying cause, as there is not an established protocol; hence, it is recommended that patients be referred to tertiary liver transplant facilities early [7]. In most cases of this disease, withdrawing the offending agent or treating the underlying condition produces clinical improvement [7]. As described by Pusl et al., ursodeoxycholic acid (UDCA) is often considered, particularly for pruritus and cholestatic symptoms, as PBC is a well‐known cause of VBDS. It is thought to improve VBDS through UDCA’s properties of stimulating hepatobiliary secretion, inhibiting apoptosis, and protecting cholangiocytes against toxic effects of hydrophobic bile acids [16]. Steroids have also been used to treat VBDS. In a case report by Mizuno et al., a 30‐year‐old male with VBDS and ATN simultaneously was started on prednisolone 40 mg on day 19 of hospitalization, with gradual improvement in bilirubin levels [7, 17]. Nevertheless, despite these medications showing promise in some cases, neither corticosteroid nor UDCA was associated with improved outcomes of biliary duct injury [8]. As shown in the prospective 10‐year cohort study by Bonkovsky et al., the factor most closely related to poor outcome was the degree of bile duct loss on biopsy [1].

## 4. Conclusion

VBDS is an uncommon disease that has been recognized to be caused by medications such as levofloxacin, meropenem, amoxicillin, and azithromycin [7]. However, supplements such as those described that our patient was taking are less commonly reported in the literature as causing VBDS. Additionally, a broad differential of causes must be considered beyond medications, as there are many other potential causes. Careful history taking was paramount in this case to obtaining the likely factor causing VBDS and stresses the importance of this to clinicians. Additionally, this case also highlights the importance of recognizing supplement‐related liver injury and unusual metabolic associations and adds to the literature an atypical case presentation of VBDS.

## Author Contributions

Edwin Mendoza, Alan Jurado, and Virali Gulla contributed to the conception of the work, data collection, and manuscript drafting. Gian Rodriguez Franco, Sherif Elhanafi, Luis Chozet, and Tamis Bright assisted with data interpretation, manuscript revision, and provided clinical oversight and critical revisions. Osvaldo Padilla contributed to pathological interpretation and review of histologic findings.

## Funding

No funding was received for this work.

## Disclosure

All authors reviewed and approved the final version of the manuscript.

## Ethics Statement

This case report was conducted in accordance with institutional and ethical standards. Written informed consent was obtained from the patient for publication of this case and the accompanying images. All efforts were made to protect patient confidentiality, and no identifying information is included in the manuscript.

## Conflicts of Interest

The authors declare no conflicts of interest.

## Data Availability

All relevant data supporting the findings of this case are included within the manuscript. Additional details are not publicly available to preserve patient confidentiality but may be provided by the corresponding author upon reasonable request.
